# Association between the expression of secreted phosphoprotein - related genes and prognosis of human cancer

**DOI:** 10.1186/s12885-019-6441-3

**Published:** 2019-12-18

**Authors:** Yaqin Tu, Cai Chen, Guorun Fan

**Affiliations:** 1 Department of Otorhinolaryngology, Union Hospital, Tongji Medical College, Huazhong University of Science and Technology, Wuhan, 430022 China; 2 Department of Endocrinology, The Central Hospital of Wuhan, Tongji Medical College, Huazhong University of Science and Technology, Wuhan, China

**Keywords:** Secreted phosphoprotein 1, Secreted phosphoprotein 2, Expression, Prognosis, Human cancer

## Abstract

**Background:**

While many studies have assessed the predictive value of secreted phosphoprotein (SPP) genes in cancer, the findings have been inconsistent. To resolve these inconsistencies, we systematically analyzed the available data to determine whether SPP1 and SPP2 are prognostic markers in the context of human cancer.

**Methods:**

The expression of SPP1 and SPP2 was assessed by Oncomine analysis. The PrognoScan database was used to assess the prognostic value of SPP1 and SPP2, with cBioPortal used to assess copy number variations. The STRING database was used to generate a Protein - Protein Interaction (PPI) network for SPP genes.

**Results:**

SPP1 was more likely to be over-expressed in breast, bladder, colorectal, head, neck, liver, lung, and esophageal cancers. SPP2 was expressed at lower levels in colorectal cancer, leukemia, liver cancer and pancreatic cancer. In addition, SPP1 and SPP2 mutations mainly occurred in cutaneous melanoma and endometrial cancer.

**Conclusions:**

Our results suggest that SPP1 and SPP2 may be effective therapeutic or diagnostic targets in certain cancers. Further research is required to confirm these results and verify the value of SPP1 and SPP2 as clinical markers of cancer prognosis.

## Background

Cancer is one of the most serious diseases threatening human health and has become a major public health problem [1]. Cancers are heterogeneous in nature; each type of cancer is associated with many unique epigenetic and genetic variations [2]. Studies exploring the processes of tumor development and those that investigate specific cancer expression profiles offer invaluable insight into both the molecular underpinnings of the disease and the potential diagnostic and therapeutic targets for use in patients [3]. Irreparable structural mutations in cells are the main cause of human cancer; these alter the DNA copy number and function of a gene at a very specific genomic location. Identifying copy number alterations is a useful approach for linking copy number alterations (CNAs) with the disease phenotype. Thus, the current study offers cell-level insight into the genetic and epigenetic changes influencing the altered biochemical processes observed in tumor cells.

Secreted phosphoprotein 1 (SPP1), also named Osteopontin (OPN), is an integrin-binding protein that is secreted from various types of cells, including macrophages, endothelial cells, and osteoclasts. In humans, SPP1 is composed of 6 introns and 7 exons, and is encoded on chromosome 4 (4q13) [4]. SPP1 is involved in multiple physiological and pathological processes. Recent studies have reported that SPP1 is significantly associated with cell growth, adherence and invasion in tumourigenesis and metastasis, and is over-expressed in lung [5], colon [6], breast [7], and prostate cancers [8]. The expression level of SPP1 correlates with tumor stage and aggressiveness, suggesting that OPN may be a diagnostic and prognostic biomarker for several cancers. On the other hand, secreted phosphoprotein 2 (SPP2) is a bone matrix protein that can bind to and inhibit the bone morphogenetic proteins (BMPs) inducing bone formation. The SPP2 gene spans approximately 27 kb at chromosome 2 (2q37.1) and encodes secreted phosphoprotein 24 kD [9]. Cancers are often associated with misregulation of the BMP signaling pathway. Previous studies have shown that SPP2 inhibits the growth of tumor cells in prostate cancer [10], pancreatic cancer [9] and hepatocellular carcinoma [11] and attenuates the growth-enhancing effects of BMP2. Thus, we hypothesize that SPP plays an oncogenic or anti-oncogenic function in different cancers. To explore the character of SPP members in cancers, oncomine platform assesses the gene expression of cancer by 86,733 microarray experiments. Furthermore, the survival of cancer patients was analyzed by PrognoScan database. The co-expression data revealed the biological function and provided insight into the potential underlying mechanism. The gene ontology enrichment by STRING is able to discover the function and regulatory mechanism of genes. Basing on many available database results pertaining to changes in gene expression or copy number, we conducted a deep analysis of alterations in SPP gene expression or copy number in the tumors of cancer patients. The goal of this analysis was to understand how the expression and mutation of these genes are associated with patient outcomes.

## Materials and methods

### Oncomine data analysis

Data sets available within the Oncomine database (https://www.oncomine.org), which compiles previously published microarray data, were employed in order to assess SPP expression patterns in different types of cancer. For each dataset we assessed comparisons of mRNA expression between tumor and normal tissue based on the following thresholds: *p*-value <1E-4, fold change > 2. We only identified the top 10% of differentially expressed genes, and using the compiled data we generated heat maps of differential SPP gene expression in different cancer types.

### Prognoscan database analysis

Using the PrognoScan database (http://dna00.bio.kyutech.ac.jp/PrognoScan/), we assessed the relationsip between SPP gene expression and survival in different cancer types, using a cox p-value threshold of < 0.05 [12].

### Protein-protein interaction (PPI) network construction

In order to better understand molecular mechanisms governing carcinogenesis, we employed the STRING database to generate a PPI network for SPP genes. We used a minimum interaction score of at least 0.4 as a cut-off when visualizing this network.

### cBioPortal database analysis

We additionally employed the open-access cBioPortal for Cancer Genomics database, which is available to assist with visualization and interpretation of large cancer genomic data (http://www.cbioportal.org/) [13, 14]. We were thereby able to review records from 215 separate studies covering 31 cancers and over 66,000 total samples. Our main parameters for exploring RNA-seq datasets with this database included SPP gene alterations (amplifications, deletions, or missense mutations) and Copy Number alterations (CNAs).

### Statistical analysis

All results are displayed with *p* values from a log-tank test. Survival curves were generated by the PrognoScan database, using a cox *p*-value threshold of < 0.05. Statistical significance of the data (*p*-values) was provided by the program.

## Results

### The expression of SPP1 and SPP2 in various cancers

**Fig. 1:**
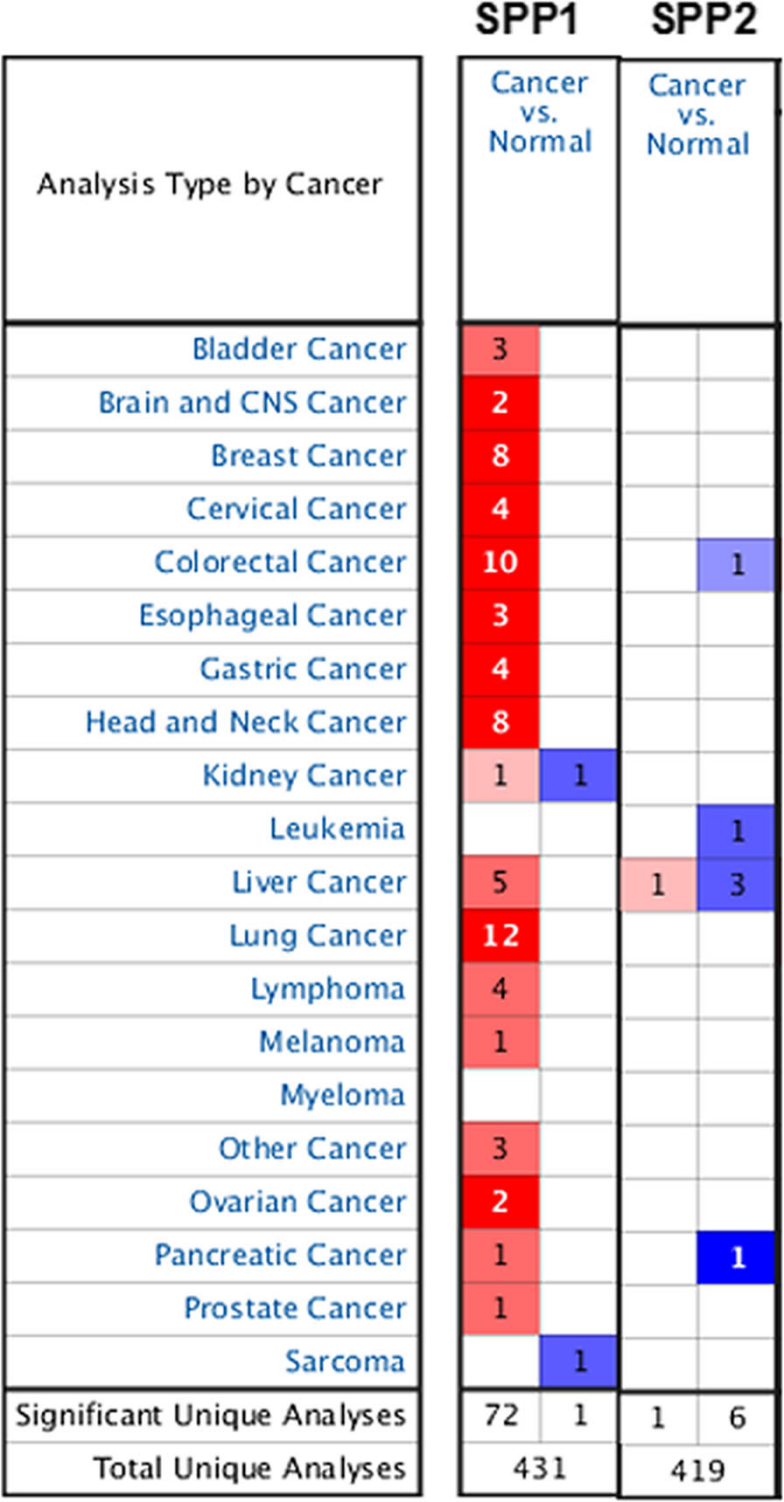
The transcription levels of SPP1 and SPP2 in different types of cancers, This graphic was generated from Oncomine, indicating the numbers of datasets with statistically significant mRNA over-expression (Red) or down-expression (Blue) of SPP1 and SPP2 in cancer versus normal tissue.The threshold was designed with following parameters: p-value of 1E-4, fold change of 2, and gene ranking of 10%

**Fig. 2:**
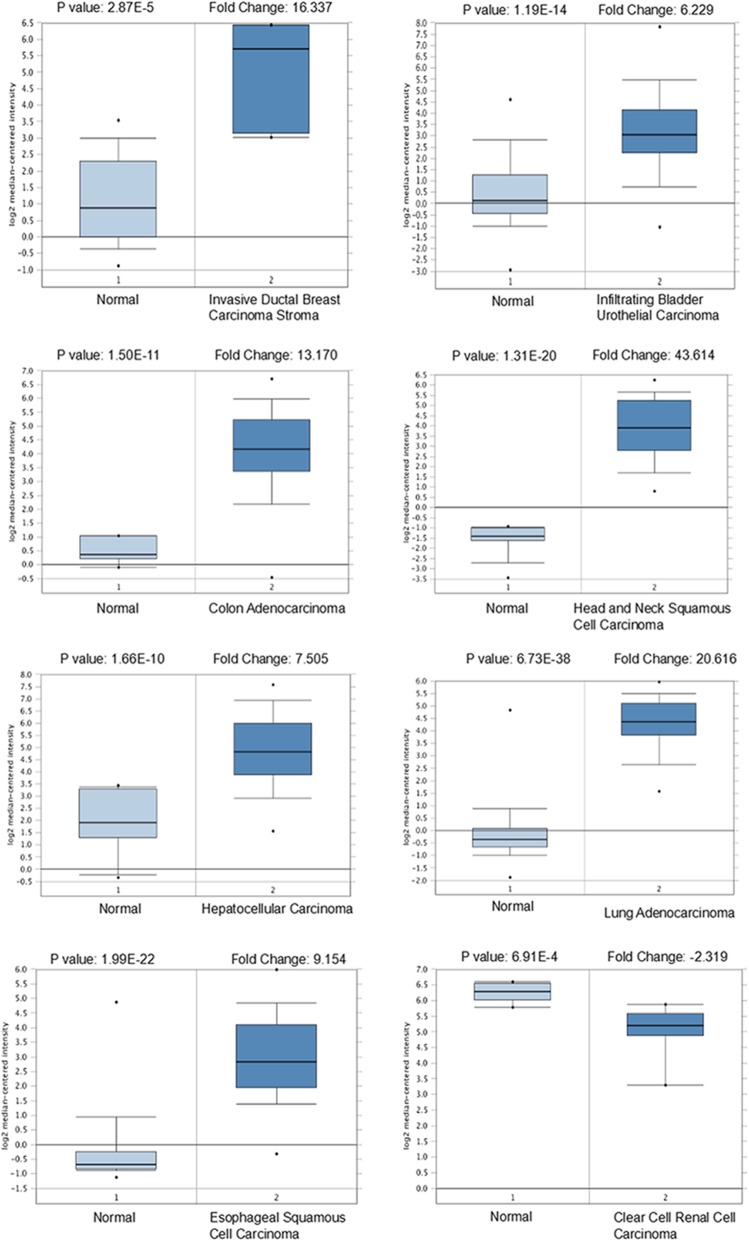
The expression level of SPP1 in different cancer types (Oncomine database), The box plot comparing specific SPP1 expression in normal (left plot) and cancer tissue (right plot) was derived from Oncomine database. The fold change of SPP1 in various types of cancers was identified from our analyses in Table 1

**Fig. 3:**
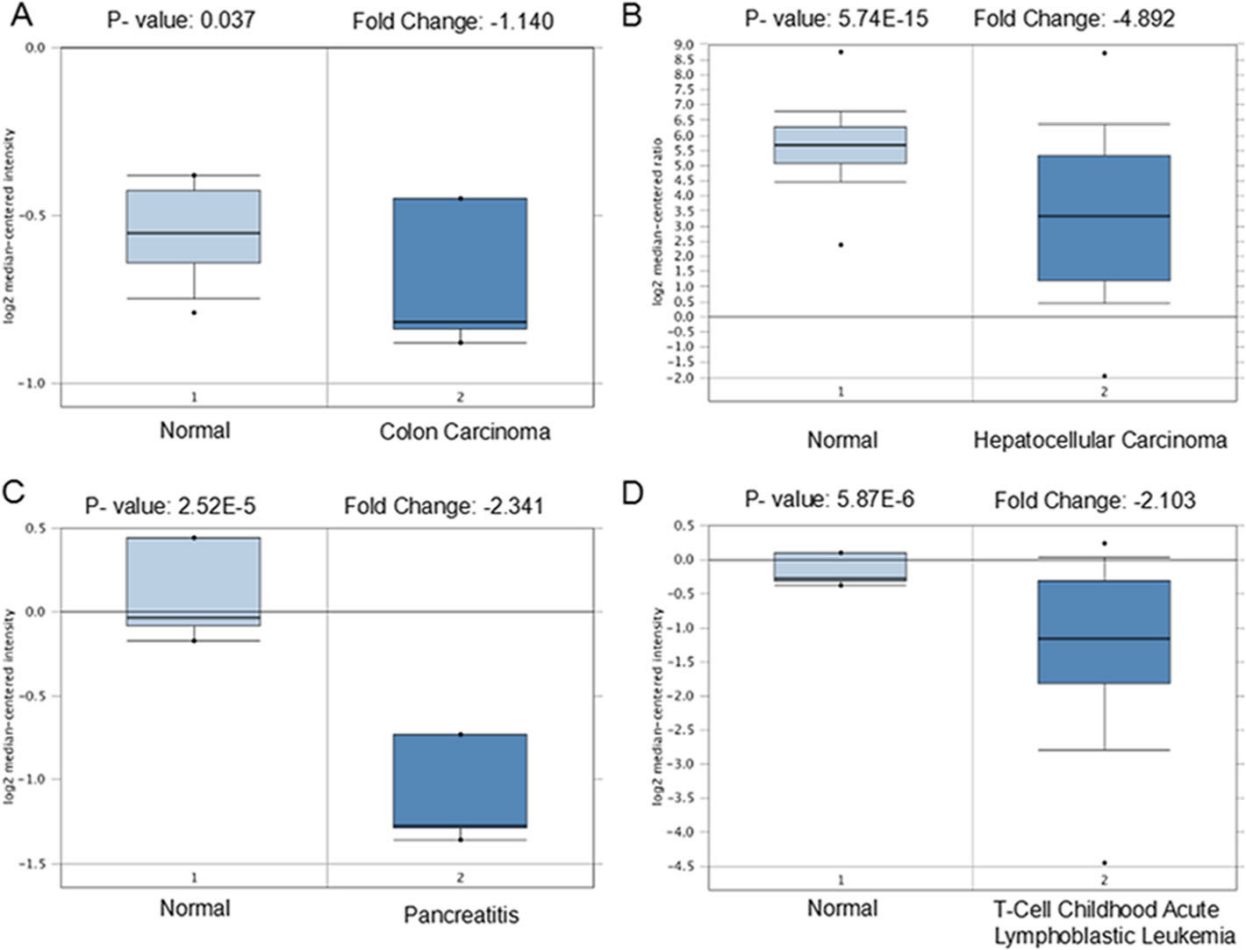
The expression level of SPP2 in different cancer types (Oncomine database), The box plot comparing specific SPP2 expression in normal (left plot) and cancer tissue (right plot) was derived from Oncomine database. SPP2 was under-expressed in colon carcinoma (**a**), hepatocellular carcinoma (**b**), pancreatic cancer (**c**), and leukemia (**d**). The fold change of SPP2 in various types of cancers was identified from our analyses in Table 2

To assess the importance of SPP in various cancers, SPP1 and SPP2 expression were analyzed in healthy and tumor tissues via the Oncomine database. We found that SPP1 was upregulated in breast, bladder, colorectal, head and neck, liver cancer, lung, and esophageal cancers, whereas decreased in kidney cancer and sarcoma (*p* < 0.05, Fig.1 and Table 1). SPP2 was under-expressed in colorectal cancer, leukemia, liver cancer and pancreatic cancer (*p* < 0.05, Fig.1 and Table 2). We additionally utilized Oncomine database to confirm SPP expression in various forms of cancer (*p* < 0.05, Fig.2 and Fig.3). We found that in certain cancers SPP1 was over-expressed, while in others it was under-expressed, suggesting that depending on the particular cancer type SPP1 may be playing a pro- or anti-oncogneic function. However, SPP2 is generally lowly expressed in tumors, suggesting that SPP2 may serve as tumor suppressor gene.

**Table 1:** SPP1 expression in cancers

Cancer	Cancer subtype	*P* value	Fold change	Sample	Reference
Lung	Lung Adenocarcinoma	6.73E-38	20.616	107	18,297,132
	Squamous Cell Lung Carcinoma	5.67E-7	60.245	203	11,707,567
colon	Colon Mucinous Adenocarcinoma	7.35E-11	35.194	105	17,615,082
	Colon Adenocarcinoma	1.50E-11	13.170	105	17,615,082
Cervix	Cervical Squamous Cell Carcinoma	1.93E-15	18.837	66	18,506,748
Brain	Glioblastoma	1.03E-6	2.346	54	16,204,036
Head-Neck	Head and Neck Squamous Cell Carcinoma	1.31E-20	43.614	54	14,729,608
	Oral Cavity Squamous Cell Carcinoma	8.04E-24	11.215	79	21,853,135
	Tongue Squamous Cell Carcinoma	2.52E-9	15.528	58	19,138,406
Ovarian	Ovarian Serous Adenocarcinoma	3.12E-5	25.623	16	14,760,385
Gastric	Gastric Cancer	2.52E-10	4.042	160	20,965,966
	Gastric Intestinal Type Adenocarcinoma	2.58E-13	15.519	96	19,081,245
Esophagus	Esophageal Squamous Cell Carcinoma	1.99E-22	9.154	106	21,385,931
Breast	Invasive Ductal Breast Carcinoma Stroma	2.87E-5	16.337	22	17,914,389
	Invasive Breast Carcinoma	7.36E-6	4.068	2136	22,522,925
	Tubular Breast Carcinoma	4.30E-19	4.813	2136	22,522,925
Bladder	Infiltrating Bladder Urothelial Carcinoma	1.19E-14	6.229	157	16,432,078
Liver	Hepatocellular Carcinoma	1.66E-10	7.505	115	19,098,997
Lymphoma	Primary Effusion Lymphoma	6.97E-8	137.979	336	15,778,709
	Centroblastic Lymphoma	2.66E-9	12.803	336	15,778,709
	Diffuse Large B-Cell Lymphoma	5.55E-18	12.096	136	19,412,164
	Unspecified Peripheral T-Cell Lymphoma	1.31E-9	8.739	60	17,304,354
Prostate	Prostate Carcinoma	1.95E-5	3.004	122	22,722,839
	Prostate Adenocarcinoma	1.23E-4	2.185	40	12,873,976
Melanoma	Cutaneous Melanoma	6.43E-8	13.322	70	18,254,958
Pancreas	Pancreatic Ductal Adenocarcinoma	4.26E-11	6.619	78	16,204,036
	Pancreatic Intraepithelial Neoplasia	3.10E-5	1.827	38	16,103,885
Sarcoma	Myxofibrosarcoma	1.01E-4	6.287	40	20,601,955
Kidney	Clear Cell Sarcoma of the Kidney	3.07E-5	−3.934	35	16,299,227

**Table 2:** SPP2 expression in cancers

Cancer	Cancer subtype	*P* value	Fold change	Sample	Reference
Breast	Invasive Lobular Breast Carcinoma	0.002	2.598	30	17,389,037
	Mucinous Breast Carcinoma	0.045	6.717	593	TCGA
Lung	Micropapillary Lung Adenocarcinoma	0.003	1.046	1537	TCGA
	Lung Adenocarcinoma	8.83E-7	2.341	246	22,080,568
Esophagus	Barrett’s Esophagus	0.023	1.230	52	16,449,976
Prostate	Prostate Carcinoma	3.36E-4	1.137	102	12,086,878
Pancreas	Pancreatic Adenocarcinoma	4.68E-5	1.033	100	TCGA
Lymphoma	T-Cell/Histiocyte-Rich Large B-Cell Lymphoma	0.011	1.220	67	18,794,340
	Diffuse Large B-Cell Lymphoma	0.009	1.132	67	18,794,340
	Burkitt’s Lymphoma	0.031	1.195	67	18,794,340
	Follicular Lymphoma	0.035	1.167	67	18,794,340
Melanoma	Skin Basal Cell Carcinoma	0.006	2.763	87	18,442,402
	Pleural Malignant Mesothelioma	0.016	2.195	54	15,920,167
	Skin Squamous Cell Carcinoma	0.048	1.906	87	18,442,402
Brain	Primary Glioblastoma	0.005	1.035	187	18,077,431
Gastric	Gastric Mixed Adenocarcinoma	0.008	1.070	90	21,447,720
Thyroid	Thyroid Gland Oncocytic Follicular Carcinoma	0.045	1.104	99	16,609,007
Liver	Hepatocellular Carcinoma	1.18E-42	−5.790	445	21,159,642
Colon	Rectal Adenocarcinoma	3.58E-4	−1.824	237	TCGA
	Colon Adenocarcinoma	6.61E-4	−1.658	237	TCGA
Ovarian	Ovarian Mucinous Adenocarcinoma	0.024	1.085	103	16,452,189
	Colon Adenocarcinoma	1.10E-5	−2.941	123	17,640,062
Pancreas	Pancreatic Carcinoma	0.003	−1.411	17	15,867,264
	Pancreatic Ductal Adenocarcinoma	1.88E-8	−1.368	78	19,260,470

### SPP expression is associated with survival in various cancers

**Fig. 4:**
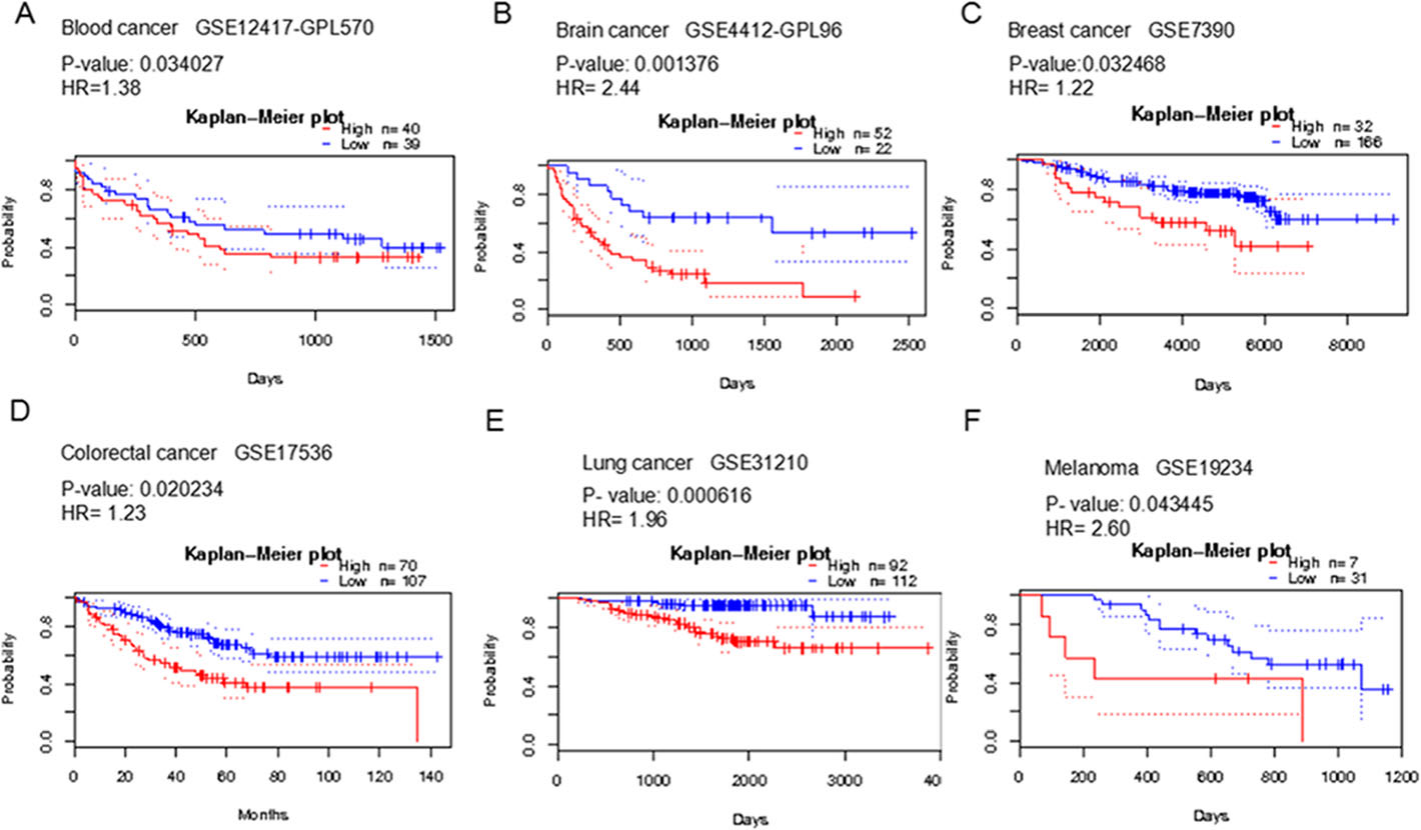
The association between the expression of SPP1 gene and prognosis in blood cancer, brain cancer, breast cancer, colorectal cancer, lung cancer and melanoma (PrognoScan database), The survival curve comparing the patient with high (red) and low (blue) expression was plotted from PrognoScan database. The survival curve comparing the patient with high (red) and low (blue) expression in blood cancer (**a**), brain cancer (**b**), breast cancer (**c**), colorectal cancer (**d**), lung cancer (**e**) and melanoma (**f**) was plotted from PrognoScan database as the threshold of cox *p*-value < 0.05

**Fig. 5:**
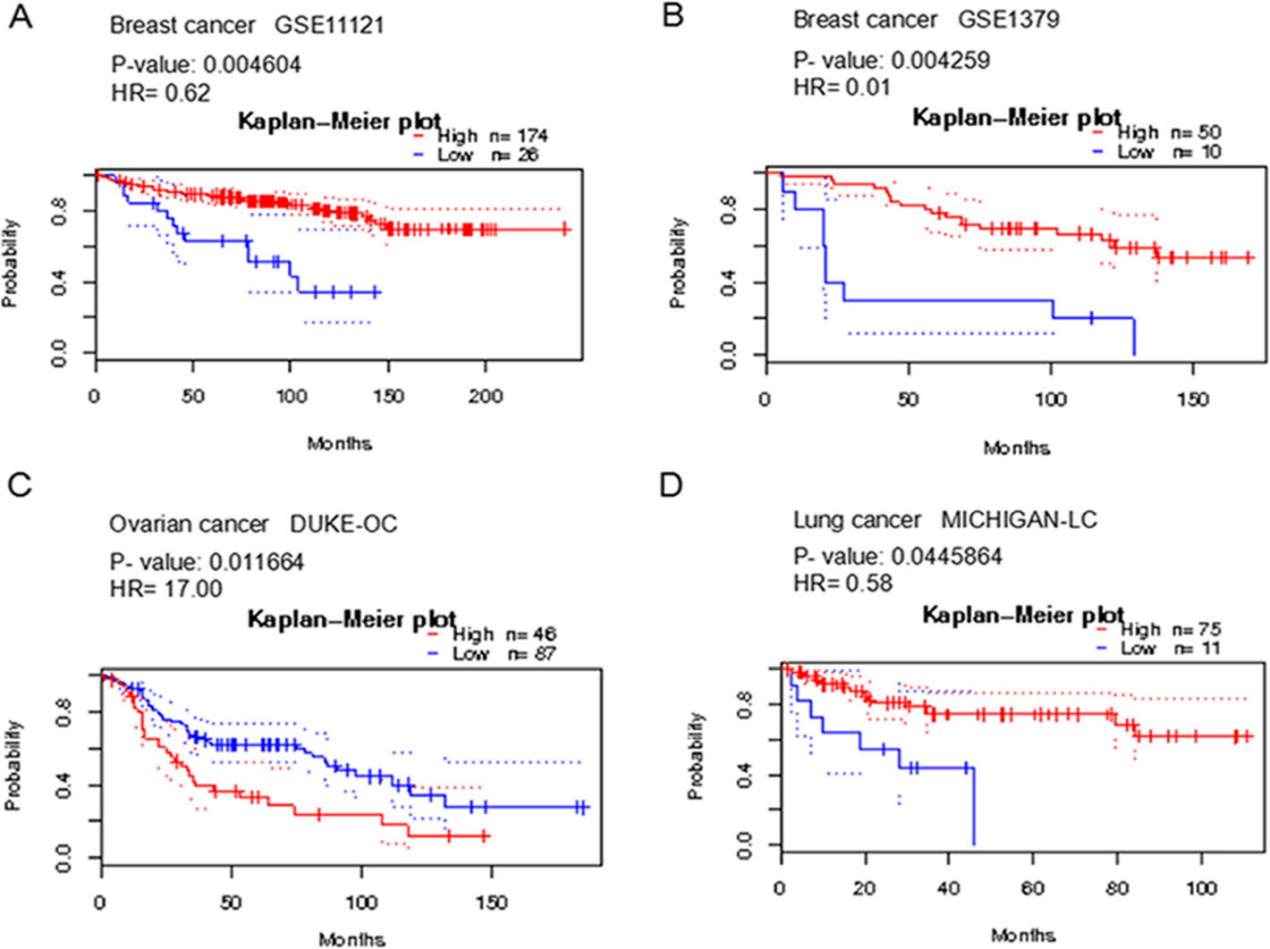
The association between the expression of SPP2 and prognosis in Breast, Ovarian and Lung cancers (PrognoScan database), The survival curve comparing the patient with high (red) and low (blue) expression was plotted from PrognoScan database. The survival curve comparing the patient with high (red) and low (blue) expression in breast cancer (**a,b**), ovarian cancer (**c**) and lung cancer (**d**) was plotted from PrognoScan database as the threshold of cox p-value < 0.05

Using a Prognostic database, we assessed the predictive link between SPP expression and patient survival in various cancer types. Patients who had a high expression of SPP1 showed poor prognosis in melanoma and in blood, brain, breast, colorectal, and lung cancer (*p* < 0.05, Fig.4, Additional file 1 and Table 3). The overexpression of SPP2 was linked with reduced survival in those with ovarian cancer, whereas it was linked with improved survival in breast and lung cancer patients (*p* < 0.05, Fig.5, Additional file 1 and Table 4).

**Table 3:** The association of SPP1 expression and the survival in cancer patients

Cancer	N	Cox *p*-value	HR	Endpoint	Dataset	Probe ID
Bladder	165	0.027573	1.24	Disease Specific Survival	GSE13507	ILMN_1651354
Blood	79	0.034027	1.38	Overall Survival	GSE12417-GPL570	209875_s_at
Brain	50	0.011310	1.32	Overall Survival	MGH-glioma	34342_s_at
	50	0.014265	1.27	Overall Survival	MGH-glioma	2092_s_at
	74	0.001376	2.44	Overall Survival	GSE4412-GPL96	209875_s_at
Breast	155	0.049236	0.83	Overall Survival	GSE9893	400
	286	0.026936	1.27	Distant Metastasis Free Survival	GSE2034	209875_s_at
	159	0.001286	1.71	Overall Survival	GSE1456-GPL96	209875_s_at
	159	0.011567	1.5	Relapse Free Survival	GSE1456-GPL96	209875_s_at
	159	0.005257	1.73	Disease Specific Survival	GSE1456-GPL96	209875_s_at
	198	0.032468	1.22	Overall Survival	GSE7390	209875_s_at
Colorectal	62	0.0411806	1.46	Overall Survival	GSE12945	209875_s_at
	177	0.0077045	1.33	Disease Specific Survival	GSE17536	209875_s_at
	177	0.0332507	1.34	Overall Survival	GSE17536	1568574_x_at
	177	0.0144318	1.45	Disease Specific Survival	GSE17536	1568574_x_at
	177	0.0202343	1.23	Overall Survival	GSE17536	209875_s_at
	145	0.00140364	1.56	Disease Free Survival	GSE17536	209875_s_at
	226	0.00135618	1.40	Disease Free Survival	GSE14333	209875_s_at
	49	0.030453	1.45	Disease Specific Survival	GSE17537	1568574_x_at
	55	0.0163347	1.31	Overall Survival	GSE17537	209875_s_at
	55	0.00149779	1.52	Disease Free Survival	GSE17537	209875_s_at
	49	0.0310856	1.38	Disease Specific Survival	GSE17537	209875_s_at
	55	0.000282164	1.72	Disease Free Survival	GSE17537	1568574_x_at
Lung	104	0.0122453	1.67	Overall Survival	jacob-00182-MSK	209875_s_at
	204	0.000247927	2.45	Overall Survival	GSE31210	209875_s_at
	204	3.04E-06	2.18	Relapse Free Survival	GSE31210	209875_s_at
	204	0.000616439	1.96	Overall Survival	GSE31210	1568574_x_at
	204	4.89E-05	1.77	Relapse Free Survival	GSE31210	1568574_x_at
	138	0.0119677	1.23	Relapse Free Survival	GSE8894	209875_s_at
	138	0.0214765	1.25	Relapse Free Survival	GSE8894	1568574_x_at
	129	0.0395	1.51	Overall Survival	GSE4573	209875_s_at
Skin	38	0.0434455	2.6	Overall Survival	GSE19234	209875_s_at

**Table 4:** The association of SPP2 expression and the survival in cancer patients

Cancer	N	Cox *p*-value	HR	Endpoint	Dataset	Probe ID
Breast	200	0.00460371	0.62	Distant Metastasis Free Survival	GSE11121	214478_at
	60	0.00425913	0.01	Relapse Free Survival	GSE1379	17,157
Colorectal	177	0.0471794	0.18	Disease Specific Survival	GSE17536	214478_at
Lung	86	0.0445864	0.58	Overall Survival	MICHIGAN-LC	U20530_at
Ovarian	133	0.0116643	17.00	Overall Survival	DUKE-OC	214478_at

### Molecular functional pathways and process of SPP1 and SPP2

**Fig. 6:**
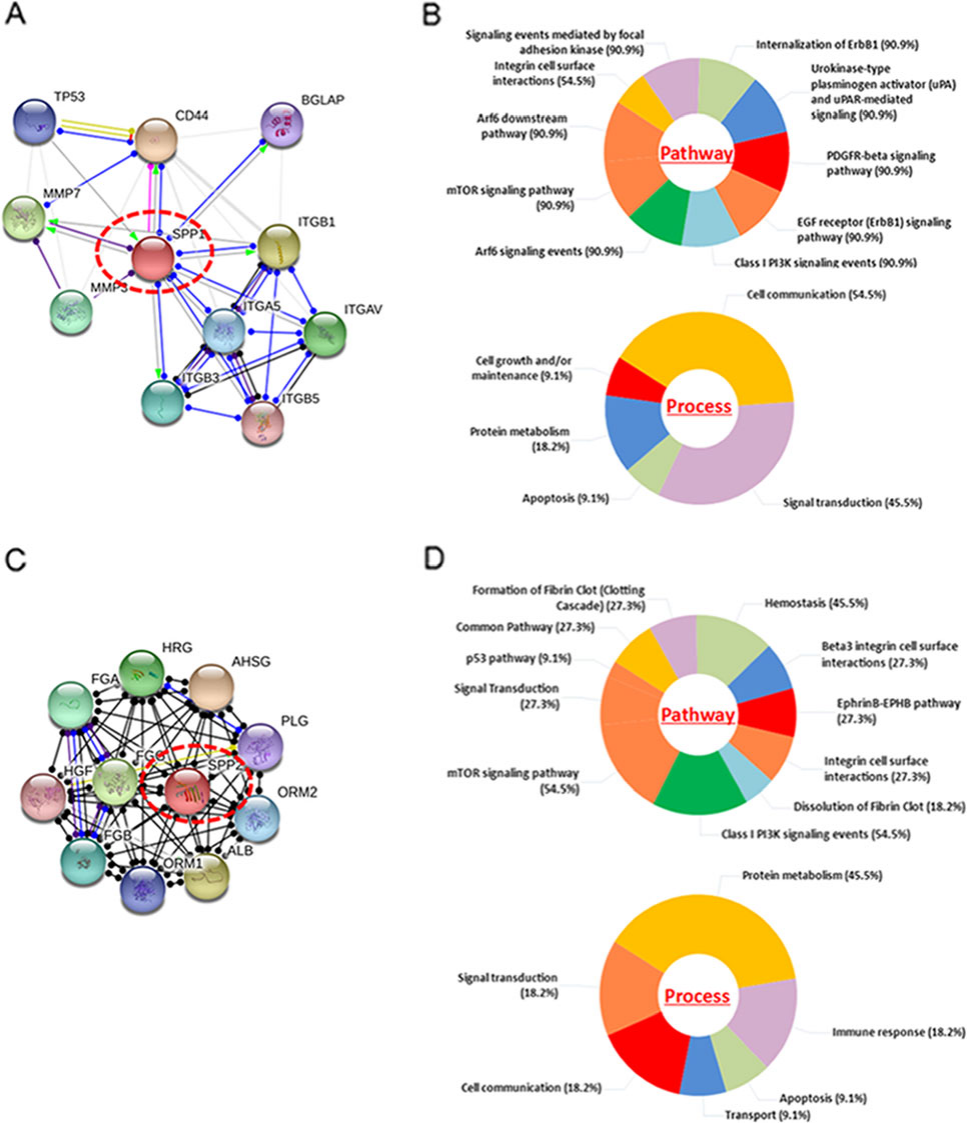
Molecular functional pathways and process of SPP1 and SPP2, Interacting nodes are displayed in colored circles using String online tools (**a, c**). Pie chart for illustration of SPP1 (**b**) and SPP2 (**d**) molecular pathways and process was analyzed by Funrich software. Most of the primary biological processes of SPP genes and 10 predicted genes were the mTOR signaling pathway and class I PI3K signaling events, along with the main process of cell growth and maintenance, signaling transduction, cell communication, and protein metabolism

We use the String database to predict the ten proteins that interact with the SPP1 and SPP2, respectively. For SPP1 these included (with the corresponding gene names): Tumor Protein P53 (TP53), Matrix Metallopeptidase 3 (MMP3), Matrix Metallopeptidase 7 (MMP7), CD44, Bone Gamma-Carboxyglutamate Protein (BGLAP), Integrin Subunit Beta 1 (ITGB1), Integrin Subunit Beta 3 (ITGB3), Integrin Subunit Beta 5 (ITGB5), Integrin Subunit Alpha 5 (ITGA5), and Integrin Subunit Alpha V (ITGAV). For SPP2 these included (with the corresponding gene names): Fibrinogen Gamma Chain (FGG), Fibrinogen Alpha Chain (FGA), Fibrinogen Beta Chain (FGB), Histidine Rich Glycoprotein (HRG), Alpha 2-HS Glycoprotein (AHSG), Plasminogen (PLG), Orosomucoid 1 (ORM1), Orosomucoid 2 (ORM2), Albumin (ALB), and Hepatocyte Growth Factor (HGF). The biological pathways and process were calculated by Funrich software, and PPI network was generated from STRING online tool (Fig.6).

### Mutations and copy number alterations of SPP genes in different cancers

**Fig. 7:**
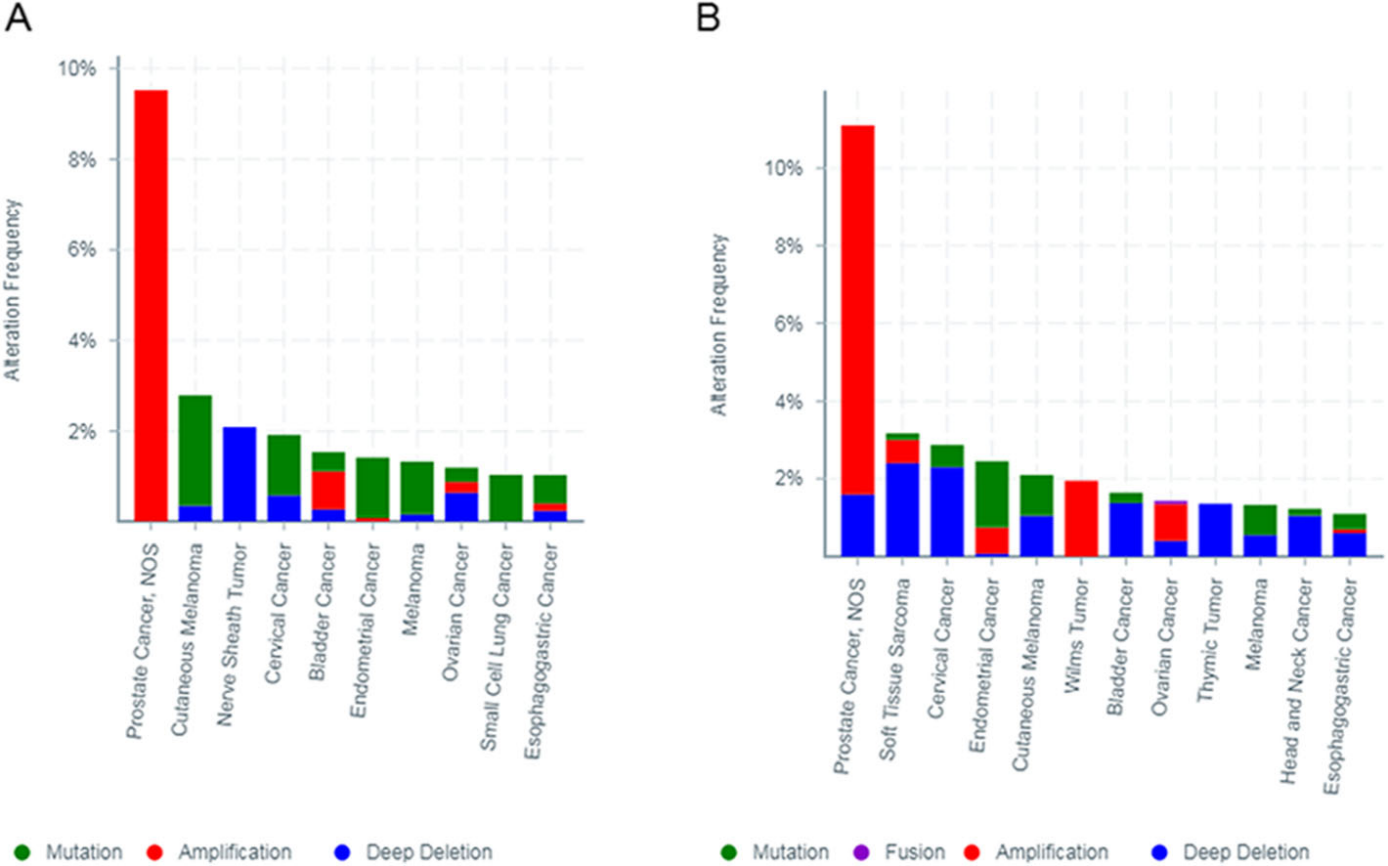
Copy number alteration of SPP genes and cancer subtypes, The alteration frequency of SPP1 (**a**) and SPP2 (**b**) was determined by cBioPortal database. The alteration frequency included deletions (blue), amplification (red), Fusion (purple) or mutation (green)

We analyzed SPP1 gene mutations and copy number alterations by via assessing 198 studies using the cBioportal tool. We observed a clear amplification pattern in prostate cancer, whereas mutations in SPP1 primarily occurred in cutaneous melanoma and endometrial cancer. The ratio of alteration ranged from 1.03 to 9.23% (Fig.7a). For SPP2 gene, an amplification pattern of interest was also observed in prostate cancer. Also, SPP2 mutation was most predominant in cutaneous melanoma and endometrial cancer. The frequency of alteration ranged from 1.06 to 10.77% (Fig.7b).

### SPP co-expression profiles in different cancers

**Fig. 8:**
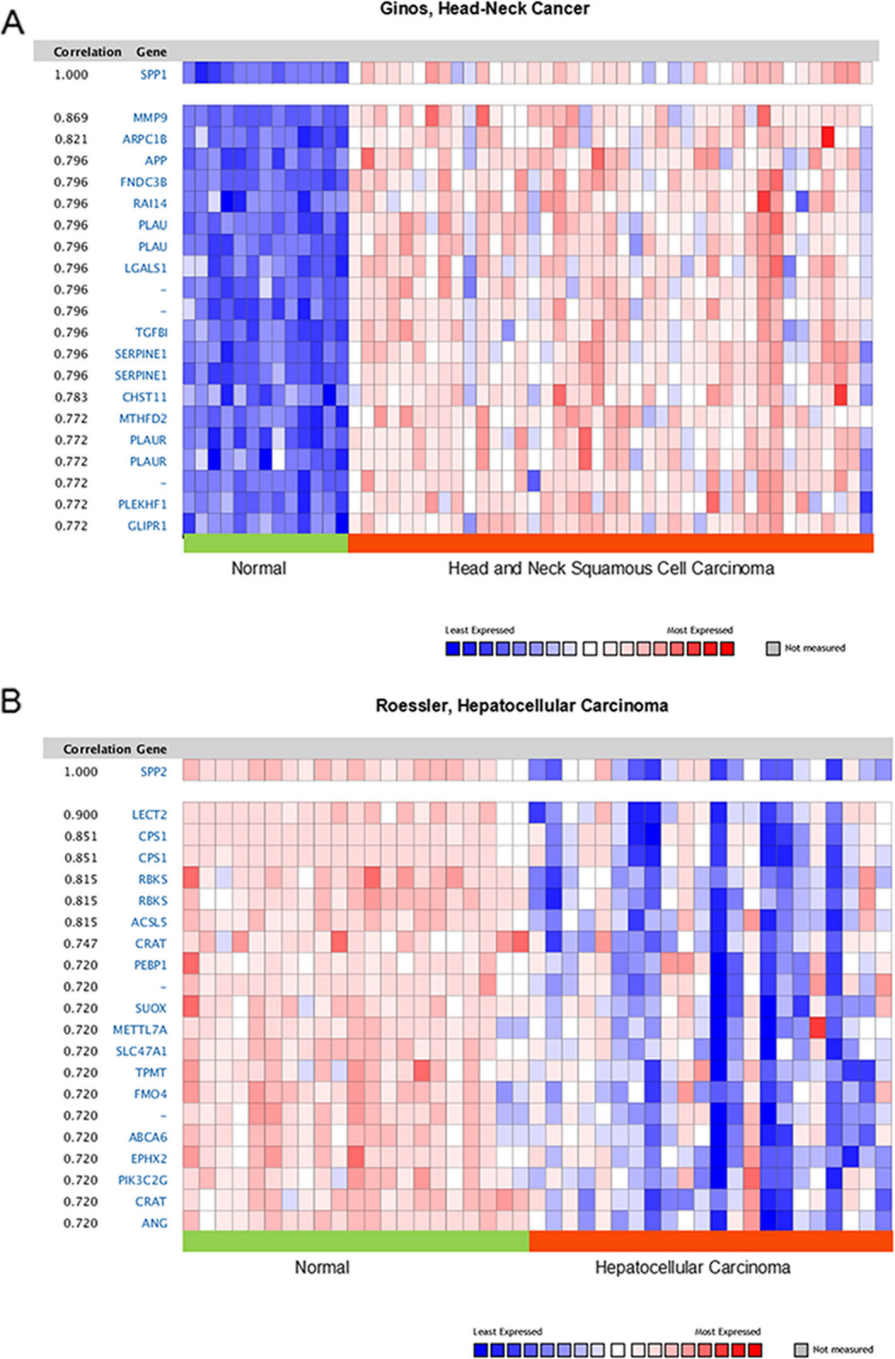
Co-expression profiles of SPP genes in different types of cancers, (**a**) SPP1 genes in head-neck squamous cell carcinoma. SPP1 is coexpressed with the indicated genes across a panel of 41 head-neck squamous cell carcinoma and 13 normal tissues. Bar length represented the significance and negative logarithm of enrichment p-value. (**b**) SPP2genes in hepatocellular carcinoma.SPP2 is coexpressed with the indicated genes across a panel of 22 hepatocellular carcinoma and 21 normal tissues. Bar length represented the significance and negative logarithm of enrichment *p*-value

Oncomine was used to analys SPP co-expression. SPP1 co-expression profiles were determined in 41 head-neck squamous cell carcinoma and 13 normal tissues. The results showed that SPP1 was over-expressed in patients with head-neck cancer, and the top 3 genes co-expressing with SPP1 were Matrix Metallopeptidase 9 (MMP9), Actin Related Protein 2/3 Complex Subunit 1B (ARPC1B) and Amyloid Beta Precursor Protein (APP) (Fig.8 a). The co-expression profile of SPP2 was identified in 22 hepatocellular carcinoma and 21 normal tissues. However, SPP2 was down-regulated in liver cancer. The top 3 genes co-expressing with SPP2 were Leukocyte Cell Derived Chemotaxin 2 (LECT2), Carbamoyl-Phosphate Synthase 1 (CPS1) and Ribokinase (Fig.8 b).

## Discussion

To date, the detailed function and role of SPP in cancer development and metastasis are poorly understood. Therefore, we assessed, for the first time, the predictive value and expression patterns of SPP in various cancers. We are not able to define particular genes as tumor suppressor genes simply based on expression levels in the absence of explicit mechanistic studies, however, tapping into extensive oncogenic databases can provide researchers with a deeper understanding of the molecular mechanisms of these genes. Analysis of the relationship between SPP1 and the prognosis of various tumors revealed that SPP1 was upregulated in breast, bladder, colorectal, head and neck, liver, lung, and esophageal cancers. Further, high levels of SPP1 gene expression were associated with a poor prognosis for these cancers. SPP1 has been reported to enhance cancer cell survival, angiogenesis, and inflammation [15], while also promoting metastasis by favoring epithelial-to-mesenchymal transition [16]. This indicates that SPP1 is a tumor-promoting gene in these cancers. On the contrary, SPP2 was found to be deregulated in colorectal cancer, leukemia, liver cancer, and pancreatic cancer. Breast and lung cancer patients with high SPP2 expression had a better prognosis. BMPs contribute to the initiation and progression of multiple cancers. SPP2 has been shown to bind to BMP-2 and inhibit tumor growth through the blockage of BMP-2 [9]. Thus, SPP2 may have great potential as a clinical therapeutic agent.

Our SPP PPI network offers valuable insight into predicted interactions and functional relationships between genes. Most of the primary biological processes of SPP genes and the 10 predicted genes were related to the mTOR signaling pathway and class I PI3K signaling events, as well as cell growth and maintenance, signaling transduction, cell communication, and protein metabolism. Given the key role of the mTOR pathway in cell growth, regulation of actin cytoskeleton, gene transcription, ribosome biogenesis, and mRNA translation, there appears to be a link between mTOR activation and cancer. PI3K activation may be important for KRAS-induced tumorigenesis. Alteration of these pathways is strongly implicated in cancer pathogenesis; thus, targeting the effectors of these pathways is a promising therapeutic approach [17].

The cBioportal analysis revealed that SPP mutations and copy number alterations mainly occurred in prostate cancer, cutaneous melanoma, and endometrial cancer. It has been reported that somatically acquired genetics, epigenetics, transcriptomics, and proteomics are the main causes of cellular carcinogenesis. These changes may lead to suppression or carcinogenesis [18]. Co-expression analyses indicated that SPP1 was co-expressed with MMP9, ARPC1B, and APP in head-neck cancer. These results indicate that these genes have similar functions in head and neck cancer, and jointly promote tumorigenesis. SPP2 was co-expressed with LECT2, CPS1, and Ribokinase in hepatocellular carcinoma. This indicates that SPP2, LECT2, CPS1, and Ribokinase exert tumor suppressive effects in hepatocellular carcinoma.

## Conclusion

To the best of our knowledge, the present study is the first to assess the importance and function of SPP family genes in the context of cancer development. Our systematic analysis of the expression and predictive value of SPP genes in various cancer types provided new insight into the heterogeneous expression of these genes in various cancers. Our findings indicate that SPP1 and SPP2 genes might play an important role in cancer progression. SPP1 appears to correlate with poor clinical outcomes, whereas SPP2 appears to be a potential prognostic marker for better survival. The inhibition or activation of SPP genes as a therapeutic approach for cancer treatment is dependent on the type of cancer to be treated. Further research is required to explore the signaling pathways and potential mechanisms of SPP genes in cancer and other diseases.

## Additional file

**Additional file 1: Figure S1.** The association between the expression of SPP genes and prognosis in cancers (Kaplan- Meier Plotter database), The prognostic value of SPP1 and SPP2 expression level in breast cancer (A, C) and ovarian cancer (B, D) was plotted from Kaplan- Meier Plotter database.

## Data Availability

The datasets generated and/or analyzed during the current study are available in the Oncomine database, [https://www.oncomine.org].

## Abbreviations

AHSG: Alpha 2-HS glycoprotein
ALB: Albumin
APP: Amyloid beta precursor protein
ARPC1B: Actin related protein 2/3 complex subunit 1B
BGLAP: Bone gamma-carboxyglutamate protein
BMPs: bone morphogenetic proteins
CAN: Copy number alterations
CPS1: Carbamoyl-phosphate synthase 1
FGA: Fibrinogen alpha chain
FGB: Fibrinogen beta chain
FGG: Fibrinogen gamma chain
HGF: Hepatocyte growth factor
HRG: Histidine rich glycoprotein
ITGA5: Integrin subunit alpha 5
ITGAV: Integrin subunit alpha V
ITGB1: Integrin subunit beta 1
ITGB3: Integrin subunit beta 3
ITGB5: Integrin subunit beta 5
LECT2: Leukocyte cell derived chemotaxin 2
MMP3: Matrix metallopeptidase 3
MMP7: Matrix metallopeptidase 7
MMP9: Matrix metallopeptidase 9
ORM1: Orosomucoid 1
ORM2: Orosomucoid 2
PLG: Plasminogen
PPI: Protein-protein interaction
SPP1: Secreted phosphoprotein 1
SPP2: Secreted phosphoprotein 2
TP53: Tumor protein P53
