# Supplementary figures and images for: Association between the expression of secreted phosphoprotein - related genes and prognosis of human cancer
Source: BMC Cancer. 2019 Dec 18;19:1230. doi: 10.1186/s12885-019-6441-3 (PMC6918603; doi:10.1186/s12885-019-6441-3)

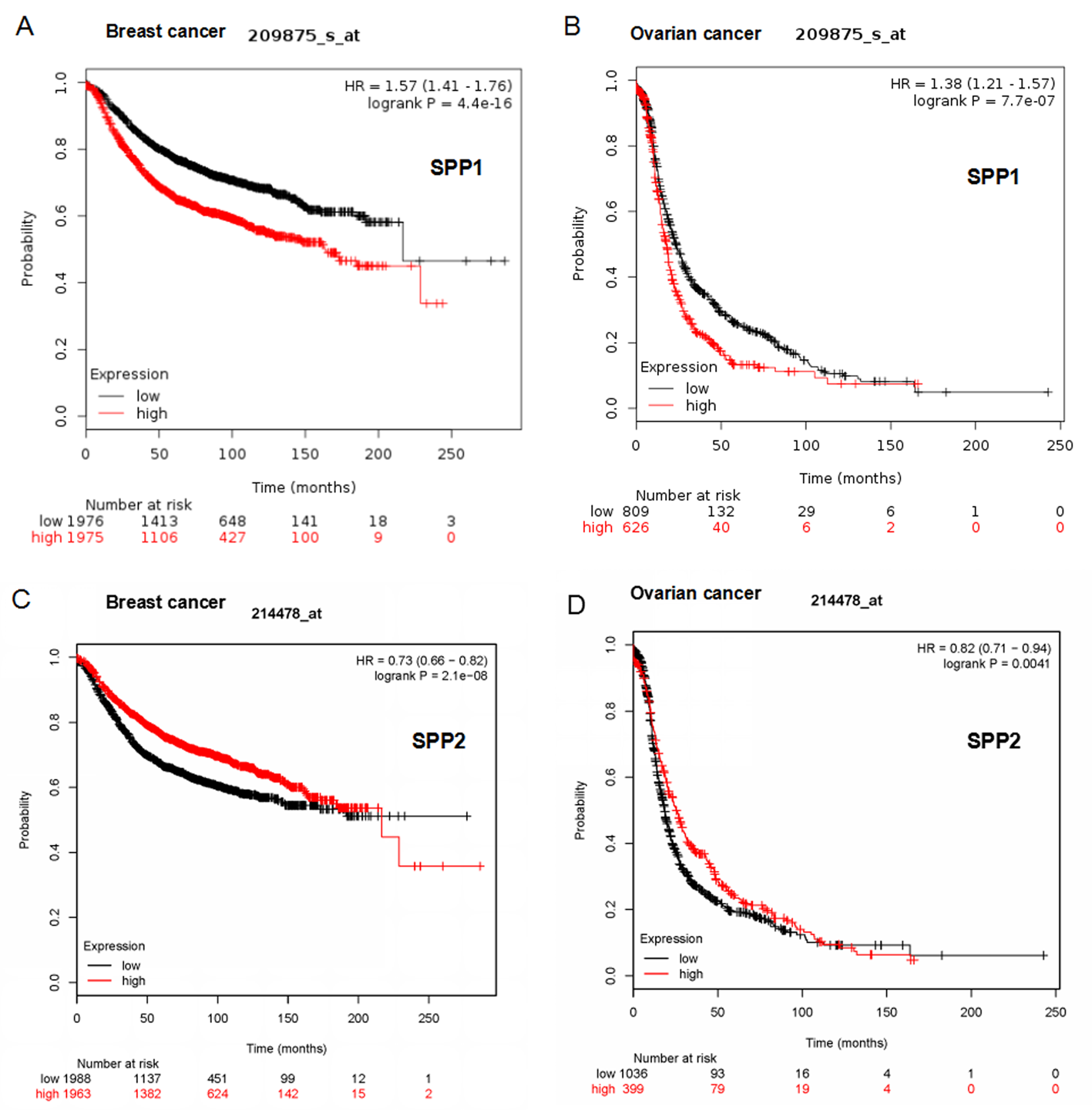

Supplement: Supplementary file 1 — Additional file 1: Figure S1. The association between the expression of SPP genes and prognosis in cancers (Kaplan- Meier Plotter database), The prognostic value of SPP1 and SPP2 expression level in breast cancer (A, C) and ovarian cancer (B, D) was plotted from Kaplan- Meier Plotter database. [file 12885_2019_6441_MOESM1_ESM.tif]
